# Complement Component C3 Participates in Early Stages of Niemann–Pick C Mouse Liver Damage

**DOI:** 10.3390/ijms21062127

**Published:** 2020-03-20

**Authors:** Andrés D. Klein, Javier González de la Vega, Silvana Zanlungo

**Affiliations:** 1 Centro de Genética y Genómica, Facultad de Medicina, Clínica Alemana Universidad del Desarrollo, Santiago 7590943, Chile; 2 Departamento de Gastroenterología, Facultad de Medicina, Pontificia Universidad Católica de Chile, Santiago 8331150, Chile; jigonzalezdelavega@uc.cl

**Keywords:** Niemann–Pick type C disease, complement cascade, liver damage, lysosomal storage diseases, foam cells, cholesterol

## Abstract

Niemann–Pick type C (NPC), a lysosomal storage disorder, is mainly caused by mutations in the *NPC1* gene. Niemann–Pick type C patients and mice show intracellular cholesterol accumulation leading to hepatic failure with increased inflammatory response. The complement cascade, which belongs to the innate immunity response, recognizes danger signals from injured tissues. We aimed to determine whether there is activation of the complement system in the liver of the NPC mouse and to assess the relationship between C3 activation, a final component of the pathway, and NPC liver pathology. Niemann–Pick type C mice showed high levels of C3 staining in the liver which unexpectedly decreased with aging. Using an inducible NPC1 hepatocyte rescue mouse model, we restored NPC1 expression for a short time in young mice. We found C3 positive cells only in non-rescued cells, suggesting that C3 activation in NPC cells is reversible. Then, we studied the effect of C3 ablation on NPC liver damage at two postnatal time points, P56 and P72. Deletion of C3 reduced the presence of hepatic CD68-positive cells at postnatal day 56 and prevented the increase of transaminase levels in the blood of NPC mice. These positive effects were abrogated at P72, indicating that the complement cascade participates only during the early stages of liver damage in NPC mice, and that its inhibition may serve as a new potential therapeutic strategy for the disease.

## 1. Introduction

Lysosomal storage disorders (LSDs) encompass approximately 70 different diseases that arise from deficiencies in lysosomal enzymes or transporters [1]. As a consequence of these genetic defects, partially degraded metabolites accumulate within lysosomes [2]. Niemann–Pick type C (NPC) disease belongs to the LSD group. Niemann–Pick type C is caused by loss of function variants in either the *NPC1* or *NPC2* genes [3,4], leading to intracellular cholesterol accumulation in every tissue. The most affected organs are the brain and the liver [5]. Niemann–Pick type C 50% of NPC patients suffer from neonatal cholestasis, jaundice, and enlarged liver and/or spleen [6]. Of these patients, 10% die from liver failure before they reach 6 months of age [7].

The complement system, which is part of the innate immune response, consists of small proteins that are capable of initiating a cytolytic response in the absence of other humoral or cellular elements. Complement activation and amplification occur on the surface of a target cell [8]. The complement cascade can be activated by the classical, the alternative or the mannose-binding lectin pathways. However, these pathways converge in the activation of a protein called C3, leading to the subsequent activation of C5 and cell lysis [9]. In the liver, hepatocytes are a main source of plasma complement proteins [10]. Nevertheless, other hepatic cell types (e.g., Kupffer cells, hepatic stellate cells, and sinusoidal endothelial cells) can also express different complement components [11]. The complement participates in several physiological and pathophysiological conditions. For instance, the complement receptor C5aR has a role in glucose release, synthesis of proinflammatory factors, and clearance of immune complexes [12]. Also, the complement cascade participates in hepatic injury in several diseases, including non-alcoholic fatty liver disease (NAFLD) [13], alcoholic steatohepatitis [14], ischemia reperfusion [15], hemorrhagic shock [16], and others. 

A recent study showed that the complement has a pathogenic role as a proinflammatory cofactor in mouse models of Gaucher disease (GD) [17]. Gaucher disease is a lysosomal disorder caused by deficiency in the lysosomal enzyme glucocerebrosidase (GCase) which causes the accumulation of glucosylceramide (GC) in the cells. This study has shown that extensive GC storage induces complement-activating IgG autoantibodies promoting C5a generation and C5aR1 activation in GD models. In turn, this response induces cellular GC accumulation, innate and adaptive immune cells recruitment and activation in GD. Indeed, pharmacological inhibition of C5aR1 prevents these inflammatory responses and increases the survival of the GD mouse. Therefore, targeting C5aR1 emerges as a potential treatment for GD patients and presumably for other LSDs.

Niemann–Pick type C patients and NPC mice models exhibit a systemic inflammatory response associated with end-organ damage and dysfunction [18]. The mechanisms that lead to tissue injury in NPC are poorly understood. Here, we studied the role of the complement cascade in NPC liver damage. We found that this pathway is highly activated during the early stages of liver disease in the NPC mouse. Interestingly, this activation seems to be reversible when the *Npc1* gene is introduced in the mutant animals, mimicking a gene therapy approach. Furthermore, we found that genetic ablation of C3 in a *Npc1*^−/−^ mouse background reduces the presence of hepatic CD68 positive cells at postnatal day 56 and prevents the increase of transaminases blood levels in NPC mice. However, these positive effects were abrogated at postnatal day 72, indicating that the complement cascade participates only in early stages of NPC liver damage.

## 2. Results

**Figure 1:**
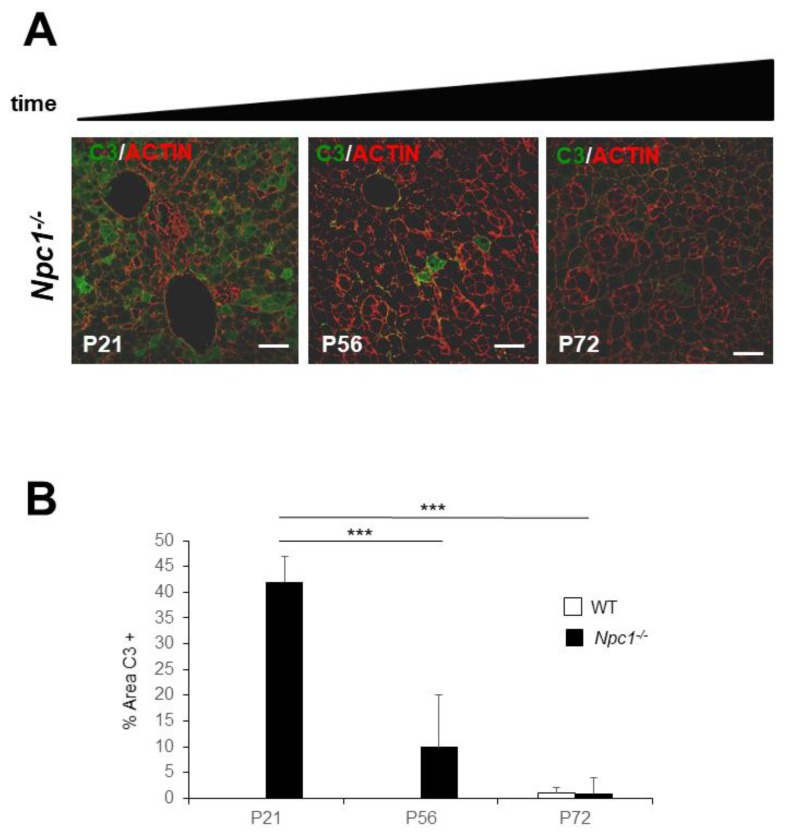
Complement component C3 (C3) deposits in Niemann–Pick type C (NPC) mouse livers decreases with aging. (**A**) Immunohistochemistry of *Npc1*^−/−^ mice livers (P21, P56, and P72 days old) showing C3 (green) and co-stained with Phalloidin-Texas red (red). (**B**) Quantification of the percentage of the area occupied by C3 positive cells at different time points in wild-type (WT) and *Npc1*^−/−^ tissues. Data are presented as means ± SD. (*n* = 5 animals/group, two-way ANOVA and Tukey’s multiple comparison post-test). *** *p* < 0.01, compared to the *Npc1*^−/−^ group. Scale bars are 50 µm.

To explore the potential activation of the complement cascade, we stained liver sections from postnatal (P) 21, 56, and 72 day old *Npc1*^−/−^ mice with antibodies against C3. To visualize the borders of the cells, we used Phalloidin-Texas Red. We found intense C3 deposition in cuboid liver cells which is the characteristic morphology of hepatocytes in young *Npc1*^−/−^ mice. This strong C3 signal decreased with aging (Figure 1A). No positive C3 staining was found in wild-type tissues (not shown, quantification in Figure 1B). 

**Figure 2:**
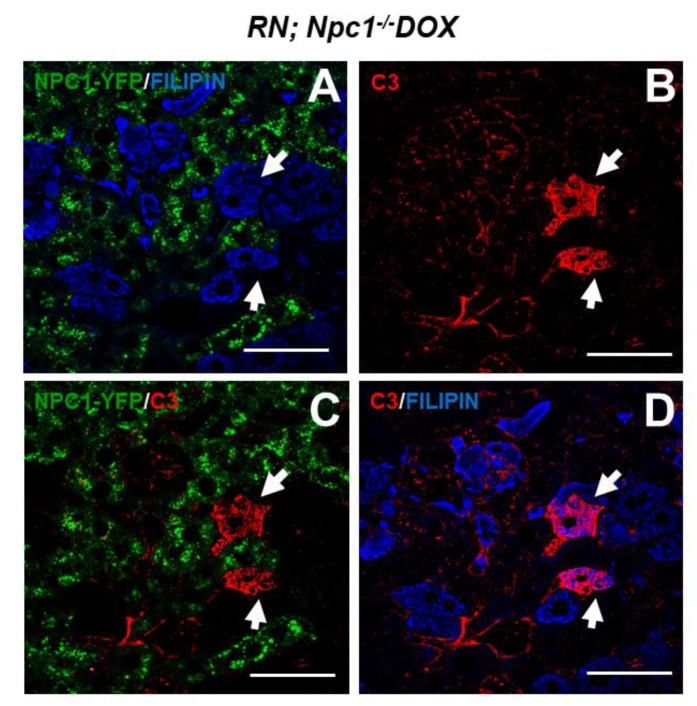
Reversible activation of the complement cascade in NPC livers. Expression of the Niemann-Pick type C1 protein fused with Yellow Fluorescent Protein (NPC1-YFP) transgene, driven by *Rosa-rtTA* in *R; N^−^ (R;N)* in the *Npc1*^−/−^ background, was induced with doxycycline from P28-33. (**A**) NPC1-YFP (green) and filipin staining (blue). (**B**) C3 staining (red). (**C**) Merged images showing NPC1-YFP (green) and C3 (red). (**D**) C3 (red) and filipin (blue) staining. Arrows indicate C3-labelled cells which were positive for filipin and negative for NPC1-YFP. Scale bars are 50 µm.

Next, we evaluated the reversibility of complement activation by inducing NPC1 expression exclusively in hepatocytes of *Npc1*^−/−^ mutant mice. To this end, we used a previously developed tetracycline-inducible transgenic system to produce the NPC1-YFP protein in hepatocytes and other visceral tissues in animals that otherwise lack NPC1 [19,20,21]. Upon doxycycline (DOX) administration to *Npc1*^−/−^ mice transgenic for ***R**OSA26-rtTA-M2* and *TRE-**N**pc1-YFP* (abbreviated RN), NPC1-YFP was produced. However, NPC1-YFP rescue in DOX-fed R;N *Npc1*^−/−^ mice was not uniform. We observed NPC1-YFP fluorescence in patches throughout the liver which fortuitously allowed us to perform mosaic analysis and compare cells that lack NPC1 with NPC1 rescued cells in the same tissue. As expected, intracellular cholesterol accumulation, visualized by filipin staining, was seen only in non-rescued liver cells (Figure 2A). Neighboring cells expressing NPC1-YFP were negative for filipin fluorescence (Figure 2A). The C3 staining revealed few C3 positive cells in the same tissue section (Figure 2B). Interestingly, these cells were non-rescued hepatocytes that also accumulated cholesterol as shown by the filipin positive signal (Figure 2C,D). These results suggest that C3 deposition is a reversible process that depends on the activity of NPC1. 

**Figure 3:**
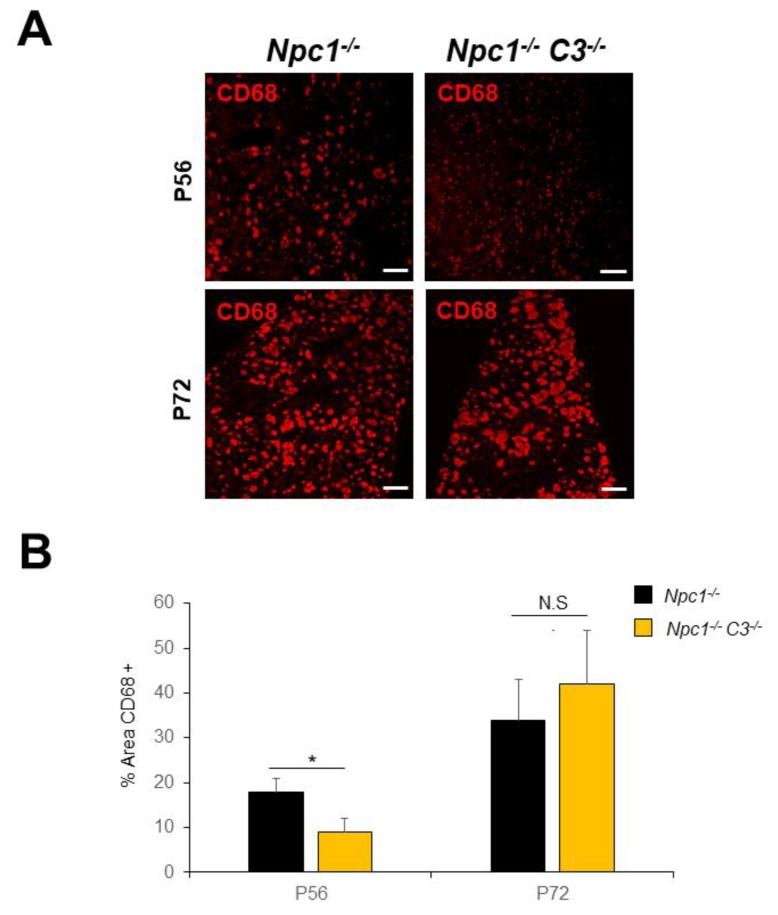
C3 ablation reduced the number of foam cells in NPC livers at P56 but not at P72. (**A**) CD68 (red) was detected in livers of P56 *Npc1*^−/−^ and dko *C3*^−/−^*Npc1*^−/−^ mice. (**B**) Quantification of the percentage of the area occupied by CD68 cells at the same time points in *Npc1*^−/−^ and dko *C3*^−/−^*Npc1*^−/−^ mice. Data are presented as means ± SD. (*n* = 5 animals/group, two-way ANOVA and Tukey’s multiple comparison post-test). * *p* < 0.01, compared to the *Npc1*^−/−^ group. Scale bars are 50 µm.

If the complement system contributes to NPC liver injury, then NPC mice lacking key components of this pathway should be protected from liver damage. Thus, we crossed *Npc1*^−/−^ mice with C3^−/−^ mice, and we assessed liver histology and physiological markers of liver damage at two different time points, P56 and P72. First, we stained liver sections from P56 days old mice with antibodies against CD68. The *C3*^−/−^ mice showed no difference in CD68 staining compared to wild-type livers (not shown). As it was previously shown, P56 *Npc1*^−/−^ mice livers had a large number of round-shaped CD68 positive cells. Indeed, CD68-positive cells occupied approximately 18% of the liver area. The dko *Npc1*^−/−^C3^−/−^ also presented foam cells. However, the area occupied by these cells was 10% of the liver sections which is significantly lower than in *Npc1*^−/−^ mice (*p* = 0.0054) (Figure 3A,B). As the disease progressed, we observed an increase in the area occupied by CD68 in *Npc1*^−/−^ mice at P72 (*p* = 0.0055). Surprisingly, at this stage dko *Npc1*^−/−^C3^−/−^ mice showed a CD68-positive area comparable to the observed in the *Npc1*^−/−^ animals (*p* = 0.2672), losing the initial protective effect. 

**Figure 4:**
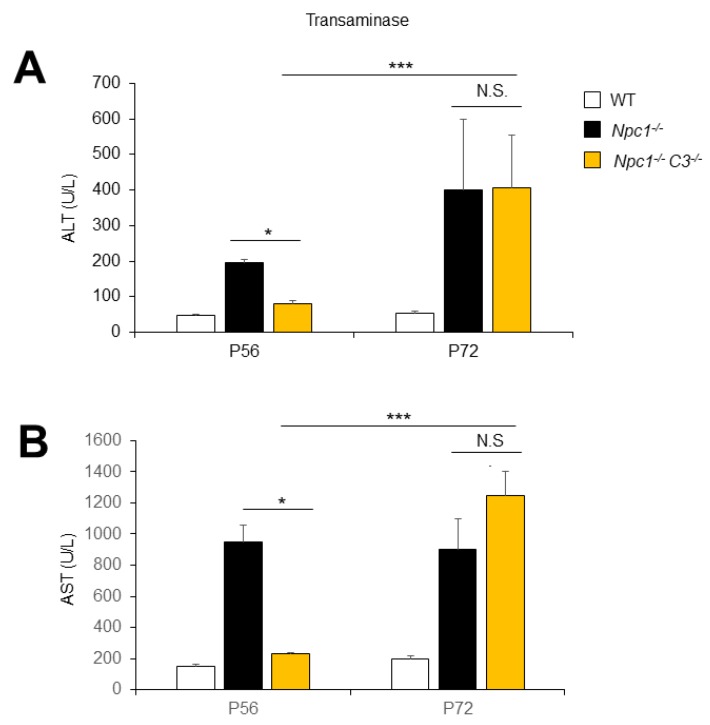
C3 ablation prevented NPC liver damage at P56 but not at P72. (**A**) Alanine and (**B**) aspartate aminotransferases (ALT and AST, respectively) were measured in P56 and P72 animals of the indicated genotypes. Data are presented as means ± SD. (*n* = 5 animals/group, two-way ANOVA and Tukey’s multiple comparison post-test). * *p* < 0.01, *** *p* < 0.001 compared to the *Npc1*^−/−^ group.

Finally, we evaluated other liver damage markers in the same animals such as the levels of alanine and aspartate aminotransferases (ALT and AST) in the serum. The *C3*^−/−^ mice showed no significant difference in ALT or AST blood levels compared to wild-type mice (*p* = 0.1449 and *p* = 0.2566, respectively). As expected, *Npc1*^−/−^ mice showed increased levels of ALT and AST compared to wild-type mice (*p* = 0.0037 and *p* = 0.0235 respectively) (Figure 4A,B). At P56, dko *Npc1*^−/−^C3^−/−^ mice showed striking reductions in serum ALT and AST levels compared to *Npc1*^−/−^ mice (*p* = 0.0161 and *p* = 0.0378, respectively) (Figure 4A,B). Congruent with previous results showing CD68 levels at P72, no differences were found in the levels of ALT and AST between *Npc1*^−/−^ and dko *Npc1*^−/−^C3^−/−^ mice (*p* = 0.9654 and *p* = 0.0846, respectively). These results demonstrate that C3 ablation prevented NPC liver damage at P56 but not at P72 and suggest that the complement cascade may serve as a new potential therapeutic target for early NPC hepatic dysfunction.

## 3. Discussion

In the present study, we showed the involvement of the complement pathway during the early stages of NPC liver damage. Our results show that the activation of this pathway decreased significantly in a short period of time. Interestingly, the genetic rescue of NPC1 seemed to reverse C3 deposits in young mice. Furthermore, the genetic ablation of C3 prevented liver damage at P56 but not at P72. These results suggest that NPC liver disease behaves as an autoimmune disorder during the early stages of the disease, opening the possibility of novel therapeutic interventions in NPC patients. 

The complement pathway participates in hepatic injury in several diseases such as ischemia reperfusion, hemorrhagic shock, non-alcoholic fatty liver disease (NAFLD), alcoholic steatohepatitis, and others [13,14,15,16]. Other studies have demonstrated the elevation of components of the complement cascade in the liver of patients with GD [17], the second most prevalent lysosomal disorder. In GD, GC triggers the activation of the complement which, in turn, induces further accumulation of GC thus generating a vicious cycle. Probably, this kind of circle does not occur in NPC, since cholesterol build up increases with age [22]. However, we showed that C3 deposits decrease with time, indicating disease-specific degenerative mechanisms. New investigations have shed some light on the pathophysiological role of the complement in GD. Mice deficient in the complement receptor C5aR1 are protected from developing the visceral aspects of the disease [17]. In NPC, genetic deletion of C1q, or of the downstream complement pathway component C3, did not significantly alter patterned neuronal loss [23]. Our results show that C3 ablation protects against liver damage in young mice, indicating tissue-specific pathophysiological regulation of complement in NPC.

Analogous to what has been described in other liver disorders [10,17], we observed C3 deposits in the liver of NPC mice. Unexpectedly, these deposits decreased as the mice aged. It is interesting to note that in healthy humans, plasma levels of complement components increase with aging [24]. Similarly, an increase in C3 protein levels has been observed with aging in mice tissues [25]. The mechanism of downregulation of C3 with time in NPC mice requires further investigation. It could be related to the natural turnover of hepatocytes and/or other compensatory changes in signaling pathways. The understanding of this regulatory mechanism might be relevant for other diseases with activation of the complement cascade.

Our work represents the first demonstration that inhibition of the complement cascade provides temporal hepato-protection in an NPC mouse model. However, the mechanisms that lead to its activation require further investigation. One possible modulator of the complement cascade in NPC disease is the Toll-Like Receptor 4 (TLR4) signaling pathway. The TLR4 signaling is increased in *Npc1*^−/−^ mice brains and livers [26,27]. Indeed, NPC mice show hepatic increments in *Tlr4* mRNA levels as early as 8 days old [27]. The TLR4 signaling leads to increments in IL-6. Interestingly, IL-6 enhances C3 transcription and secretion in a rat hepatoma cell line [28]. Thus, it seems probable that TLR4 signaling may contribute to the early increments in C3 deposits observed in NPC mice livers.

Previous studies have characterized NPC inflammation; however, whether liver inflammation is promoting tissue injury or protecting from it has not yet been clearly established. There are few reports describing the relevance of anti-inflammatory molecules in NPC liver dysfunction. For instance, the use of rapamycin, an FDA-approved immune suppressor, doubled the life span of NPC mice with enhanced visceral disease [29,30]. The hepatic engagement of other pro-inflammatory pathways has been studied as well. It was determined that TNF-α is increased in NPC mouse livers. The physiological relevance of TNF-α signaling for NPC liver damage was assessed by genetic deletion and with blocking antibodies. Both strategies led to delayed progression of NPC liver disease in mice [31,32], supporting the use of anti-inflammatory drugs to treat the hepatic symptoms of the disease. 

Our experiments suggest that restoring cholesterol efflux in NPC hepatocytes, by genetically reintroducing *Npc1*, can reverse C3 deposits. Another strategy to induce cholesterol efflux pharmacologically is the use 2-hydroxypropyl-β-cyclodextrin (cyclo). Cyclo injections are one of the most effective ways to treat NPC mice and cats [33,34,35]. Moreover, an 18 month open-label phase 1/2a trial with 14 NPC participants and monthly lumbar IT cyclo delivery showed slowing of disease progression [36]. Even one single cyclo injection at P7 induces cholesterol efflux from almost every tissue and extends NPC mouse lifespan. Cyclo injections improve NPC liver damage that is correlated with a reduction in several inflammatory markers, such as TLR4, as early as 24 h post injection [37]. Thus, it is feasible that cyclo treatments could be protecting from NPC liver disease by decreasing the activation of the complement cascade.

The complement cascade can directly activate hepatic macrophages [38,39,40]. In the past, we assessed the role of foam cells on NPC liver damage with gadolinium chloride (GdCl_3_), a well-known Kupffer/foam cell inhibitor. Interestingly, GdCl_3_ treatment decreased the levels of CD68-positive cells in the liver to a similar extent as the C3 deletion at P56. In both cases there is a normalization of transaminase levels in the serum of NPC mice [21]. This study showed that targeting foam cells with gadolinium improved NPC liver disease. Further studies are required to determine the effect of targeting foam cells on complement activation.

In summary, our results showed that the complement cascade participates during early stages of NPC liver damage, and its inhibition may serve as a new potential therapeutic strategy. Interestingly, in 2007 the US Food and Drug Administration approved anti-C5 blocking therapies for treating paroxysmal nocturnal hemoglobinuria and more recently for atypical hemolytic uremic syndrome [41,42]. Therefore, it would be interesting to study the relevance of the complement in liver disease of NPC patients, since these antibodies could be potentially beneficial for the treatment of NPC patients.

## 4. Materials and Methods

### 4.1. Mouse Strains and Genotyping

All procedures were in compliance with national regulations set by Stanford’s Administrative Panel on Laboratory Animal Care. We used *Npc1*^−/−^ mice in the FVB background which developed a disease indistinguishable from that in the BALB/c background [15,16]. Transgenic ROSA26-rtTA-M2; TRE-Npc1-YFP, *Npc1*^−/−^ mice for liver rescue, were in the FVB genetic background. The breeding strategy to generate the strains and the genotyping protocols has been previously reported [16]. The experiments were performed at Stanford University School of Medicine. The C3^−/−^ mice were in the C57BL/6 genetic background and the genotyping protocol has been previously described [19]. The dko *Npc1*^−/−^ C3^−/−^ mice were in an FVB-C57BL/6 mixed background. Protocols were performed according to accepted criteria for the humane care of experimental animals and were approved by the review board for animal studies of Stanford University (APLAC protocol #10412, 09/22/2011). Five male animals per genotype were used.

### 4.2. Doxycycline Treatments

Doxycycline (Sigma Chemicals Co., St. Louis, MO, USA) was administered in the drinking water at 2 mg/mL in 5% sucrose, every 3 days from P28–P33. 

### 4.3. Immunofluorescence, Filipin Staining, and Image Quantification

Procedures were performed as previously described [20]. Primary antibodies used were rat anti-C3 (Cederlane, Burlington, Ontario, Canada), chicken anti-GFP (Aves, Tigard, OR, USA), and rat anti-CD68 (Abd serotec, Düsseldorf, Germany). Secondary Alexa antibodies and Phalloidin Texas-Red were purchased from Invitrogen (Carlsbad, CA, USA). Filipin complex was obtained from Sigma (Sigma Chemicals Co., St. Louis, MO, USA).

### 4.4. Liver Panel

The ALT and AST analyses on mouse blood samples were performed by Stanford University Veterinary Service Center. 

### 4.5. Statistics

Statistical analyses were performed using GraphPad Prism 6 (version 6, Graphpad Software Inc., San Diego, CA, USA). The ANOVA followed by Tukey’s multiple comparison tests were used for statistical comparisons among the groups. Data in graphs are shown as mean ± SEM.
